# The complete mitochondrial genome of *Takydromus amurensis* (Squamata: Lacertidae)

**DOI:** 10.1080/23802359.2016.1155091

**Published:** 2016-03-28

**Authors:** Wei-Wei Ma, Huan Liu, Wen-Ge Zhao, Peng Liu

**Affiliations:** College of Life Science and Technology, Harbin Normal University, Harbin, P.R. China

**Keywords:** Lacertidae, mitogenome, phylogenetic tree, *Takydromus amurensis*

## Abstract

The complete mitogenome sequence of *Takydromus amurensis* (Squamata: Lacertidae) is determined using long PCR for the first time in this study. It is a circular molecule of 17 333 bp in length (GenBank accession number: KU641018). Similar to the most other lizards, the complete mtDNA sequence of *T. amurensis* contained two rRNA genes (12S rRNA and 16S rRNA), 22 tRNA genes, 13 protein-coding genes (PCGs) and a control region (D-loop). The nucleotide composition was 31.23% A, 26.06% C, 13.91% G and 28.8% T. Mitochondrial genomes analyses based on NJ method yield phylogenetic trees, including 14 reported lizards belonging to three families (Lacertidae, Gekkonidae and Agamidae). These molecular data presented here provide a useful tool for systematic analyses of genus *Takydromus*.

The interrelationships and phylogeny evolution of East Asian grass lizards of the genus *Takydromus* (Lacertidae) have been reported with morphological characters and DNA sequences (Arnold 1997; Lin et al. 2002; Ota et al. 2002; Lin et al. 2006). In recent years, with the development of mitochondrial DNA (mtDNA) molecular marker technology, the complete mitochondrial genome of *T. wolteri* and *T. sexlineatus* is sequenced (Yu & Ji 2013; Qin et al. 2015). In order to perform a systematic analysis with these molecular data, the complete mitochondrial genome of the Heilongjiang grass lizard *T. amurensis* is determined for the first time with a muscle sample using a primer walking strategy and the long and accurate PCR in this paper. This small multiple-clutched oviparous insectivorous lizard is mainly found in Northeast China, Russia and Korean Peninsula (Zhao et al. 1999). The specimen was collected from Changbai Mountain in Jilin Province of China (42°36′18.5″, 127°50′44.8″E) and was stored in Zoological and Botanical Specimen Museum of Harbin Normal University (its accession number is HRB1506080).

The obtained complete sequence of *T. amurensis* is 17 333 bp long and contains two rRNA genes (12S rRNA and 16S rRNA), 22 tRNA genes, 13 protein-coding genes (PCGs) and a control region (CR; D-loop). This structural arrangement corresponds to that of the typical lizard mitogenome (Boore 1999; Böhme et al. 2007). The nucleotide composition is 31.23% A, 26.06% C, 13.91% G and 28.8% T. The accurate annotated mitochondrial genome sequence was submitted to GenBank with accession number KU641018.

Within the mitochondrial genome of *T. amurensis*, there are 11 reading frame overlaps (share 1–10 nucleotides) and nine intergenic spacers (range from 1 to 3 bp). Nine genes (*ND6* and eight tRNA genes) are encoded on the light strand (L-strand) and the remainders are on the heavy strand (H-strand). All the 13 PCGs begin with ATG as start codon. *ND1*, *ATP8*, *ATP6*, *ND3*, *ND4L*, *ND5* and *CYTB* genes are terminated with TAA as stop codon; *COII*, *COIII* and *ND4* end with a single stop nucleotide T, *ND2* ends with TAG, *COI* ends with AGG, and *ND6* ends with AGA. The 22 tRNA genes with the size ranging from 64 to 73 bp are interspersed along the whole genome. The sequence length of the 12S and 16S rRNA is 950 and 1531 bp, respectively, D-loop region is 1953 bp. In the WANCY cluster of tRNA genes, a 26 bp sequence is considered as the putative L-strand replication origin, OL.

Mitochondrial genomes analyses based on MP, ML and NJ yielded identical phylogenetic trees, including 14 reported lizards belonging to three families (Lacertidae, Gekkonidae and Agamidae) (Figure 1). It appeared that *T. amurensis* and *T. wolteri* formed a monophyletic group as they are similar in the morphology and distribution (Zhao et al. 1999), they belong to the same group as *T. sexlineatus*. This study will facilitate the further research on comparing the genetic structure of the related species such as *T. sylvaticus* (Tang et al. 2014) and systematic analyses of the genus *Takydromus*.

**Figure 1.**
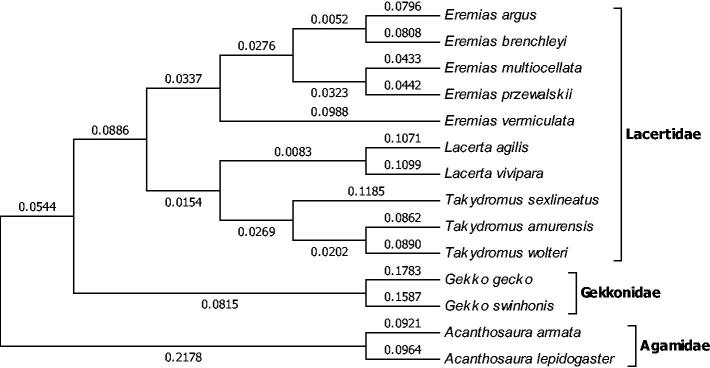
Phylogenetic tree generated using the neighbour-joining method based on complete mitochondrial genomes of some species of Lacertilia. *Eremias argus* (NC_016755), *Eremias brenchleyi* (NC_011764), *Eremias multiocellata* (NC_025304), *Eremias przewalskii* (NC_0259294), *Eremias vermiculata* (NC_025320), *Lacerta agilis* (KC990830), *Lacerta vivipara* (NC_026867), *Takydromus sexlineatus* (NC_022703), *Takydromus amurensis* (KU641018), *Takydromus wolteri* (NC_018777), *Gekko gecko* (NC_007627), *Gekko swinhonis* (NC_018050), *Acanthosaura armata* (NC_014175) and *Acanthosaura lepidogaster* (KR092427).
